# Association study of genetic variants of pro-inflammatory chemokine and cytokine genes in systemic lupus erythematosus

**DOI:** 10.1186/1471-2350-7-48

**Published:** 2006-05-23

**Authors:** Elena Sánchez, José M Sabio, José L Callejas, Enrique de Ramón, Rosa Garcia-Portales, Francisco J García-Hernández, Juan Jiménez-Alonso, Ma Francisca González-Escribano, Javier Martín, Bobby P Koeleman

**Affiliations:** 1 Instituto de Parasitología y Biomedicina López-Neyra, CSIC, Granada, Spain; 2 Servicio de Medicina Interna. Hospital Virgen de las Nieves, Granada, Spain; 3 Servicio de Medicina Interna. Hospital Clínico San Cecilio, Granada, Spain; 4 Servicio de Medicina Interna. Hospital Carlos-Haya, Málaga, Spain; 5 Servicio de Reumatología, Hospital Virgen de la Victoria, Málaga, Spain; 6 Servicio de Medicina Interna, Hospital Virgen del Rocio, Sevilla, Spain; 7 Servicio de Inmunología. Hospital Virgen del Rocío, Sevilla, Spain; 8 Department of Biomedical Genetics, Utrecht University Medical Centre, Utrecht, The Netherlands

## Abstract

**Background:**

Several lines of evidence suggest that chemokines and cytokines play an important role in the inflammatory development and progression of systemic lupus erythematosus. The aim of this study was to evaluate the relevance of functional genetic variations of *RANTES, IL-8*, *IL-1α*, and *MCP-1 *for systemic lupus erythematosus.

**Methods:**

The study was conducted on 500 SLE patients and 481 ethnically matched healthy controls. Genotyping of polymorphisms in the *RANTES, IL-8*, *IL-1α*, and *MCP-1 *genes were performed using a real-time polymerase chain reaction (PCR) system with pre-developed TaqMan allelic discrimination assay.

**Results:**

No significant differences between SLE patients and healthy controls were observed when comparing genotype, allele or haplotype frequencies of the *RANTES*, *IL-8*, *IL-1α*, and *MCP-1 *polymorphisms. In addition, no evidence for association with clinical sub-features of SLE was found.

**Conclusion:**

These results suggest that the tested functional variation of *RANTES*, *IL-8, IL-1α*, and *MCP-1 *genes do not confer a relevant role in the susceptibility or severity of SLE in the Spanish population.

## Background

Systemic lupus erythematosus (SLE) is a chronic and systemic autoimmune disease with a complex pathogenesis involving multiple genetic and environmental factors. The disease is characterized by autoantibody production, abnormalities of immune-inflammatory system function and inflammatory manifestation in several organs. Although the pathogenesis of SLE is unknown, the increased concordance of SLE in monozygotic versus dizygotic twins and familial clustering provide evidences for the role of genetic factors in this disorder [1]. However, the genetic background of SLE is thought to be complex and involves multiple genes encoding different molecules with significant functions in the regulation of the immune system [1-4]. Among the genetic factors believed to influence susceptibility to SLE, the major histocompatibility complex (MHC) alleles show the most significant association. Importantly, several recent studies show that non-HLA genes play a role in the development of SLE [1-4]. In this respect, there are several lines of evidence that chemokines and cytokines play an important role in the inflammatory development and progression of autoimmune diseases as SLE [5-7]. Furthermore, it has been show that SLE patients show an up-regulation of inflammatory molecules [8,9].

Regulated upon activation, normal T cell expressed and secreted (*RANTES*), interleukin 8 (*IL-8*) and monocyte chemoattractant protein-1 (*MCP-1*) are involved in the physiology and pathophysiology of acute and chronic inflammatory processes, by recruitment of monocytes, T lymphocytes and eosinophils to sites of inflammation [10,11]. Substantial evidence suggest that *IL-8 *and *MCP-1*, contribute to kidney injury in the glomerulonephritis of SLE, through glomerular leukocyte infiltration [12,13]. Serum levels of these inflammatory chemokines (*RANTES*, *IL8 *and *MCP-1*) are significantly higher in SLE patients than in control subjects, and correlated significant with SLEDAI score, suggesting a role in the pathogenesis of the disease [9]. As a consequence of renal disease, increased urine MCP-1 and urine IL-8 (uMCP-1, uIL-8) levels can be detected in SLE patients during active renal disease [14]. Interestingly some genetic variants within regulatory regions of these genes can affect the transcriptional activity and subsequent protein expression in human. For, *RANTES *the SNPs -403 G/A (rs2107538) and R3 (rs2306630) T/C, for *IL-8 *-353 T/A (rs4073) and for +781 C/T (rs2227306) and *MCP-1 *-2518 G/A (rs1024611) have been correlated to mRNA and or protein expression [15-17].

In addition to these three genes, *IL-1α *also constitutes a strong candidate gene for SLE, since it is a proinflammatory cytokine that plays and important role in initiating and modulating the immune responses. There is a functional polymorphism in the promoter region of *IL-1α *gene at position -889 C/T (rs1800587), and the -889 C homozygous genotype has been associated with significantly lower transcriptional activity of the *IL-1α *gene and lower levels of *IL-1α *in plasma compared with the TT genotype [18].

Overall, the chemokines *RANTES*, *IL-8*, *MCP-1*and cytokine *IL-1α *are strong candidate genes for which genetic association studies can shed light on the underlying mechanisms causing the immune dysregulation, such as inappropriate T cell activation or trafficking in SLE.

Therefore, the aim of this work was to test for association of the reported functional polymorphisms in *RANTES*, *IL-8*, *MCP-1 *and *IL-1α *with SLE susceptibility.

## Methods

### Patients

Peripheral blood samples were obtained after written informed consent from 500 SLE patients meeting the American College of Rheumatology (ACR) criteria for SLE [19]. These patients were recruited from five Spanish hospitals: Hospital Virgen de las Nieves and Hospital Clinico (Granada), Hospital Virgen del Rocio (Seville) and Hospital Carlos-Haya and Hospital Virgen de la Victoria (Malaga). Similarly, blood was taken from 481 blood bank and bone marrow donors of the corresponding cities that were included as healthy individuals. Both patient and control groups were of Spanish Caucasian origin and were matched for age and sex. Eighty seven percent of the SLE patients were women, the mean age of SLE patients at diagnosis was 43 ± 13.3 years and the mean age at disease onset of SLE symptoms was 32 ± 15 years. The SLE clinical manifestations studied were articular involvement (76%), renal affectation (37%), cutaneous lesions (62%), hematopoietic alterations (73%), photosensivity (51%), neurological disease (17%) and serositis (28%). The study was approved by all local ethical committees from the corresponding hospitals.

### Genotyping

For all the considered SNPs, samples were genotyped using a pre-developed TaqMan allelic discrimination assay. Table 1 shows the part number and reference of each SNP (Applied Biosystems, Foster City, CA, USA). PCR was carried out with mixes consisting of 8 ng of genomic DNA, 2.5 μl of Taqman master mix, 0.125 μl of 20x assay mix and ddH_2_O up to 5 μl of final volume. The following amplification protocol was used: denaturation at 95°C for 10 min, followed by 40 cycles of denaturation at 92°C for 15 sec and annealing and extension at 60°C for 1 min. After PCR, the genotype of each sample was attributed automatically by measuring the allelic specific fluorescence on the ABI PRISM 7900 Sequence Detection Systems using the SDS 2.2.2 software for allelic discrimination (Applied Biosystems, Foster City, CA, USA).

**Table 1:** Taqman probes part number used for genotyping.

Polymorphisms	Part number
*RANTES *-403 G/A (rs2107538)	C_15874407_10
*RANTES *R3 C/T (rs2306630)	C_26625663_10
*IL-8 *-353 A/T (rs4073)	C_11748116_10
*IL-8 *+781 C/T (rs2227306)	C_11748169_10
*IL-1α *-889 C/T (rs1800587)	C_9546481_20
*MCP-1 *-2518 G/A (rs1024611)	C_2590362_10

### Statistic analysis

Allele and genotype frequencies were obtained by direct counting. Hardy-Weinberg equilibrium and statistical analysis to compare allelic and genotypic distributions were performed using the chi-square test. Odds ratio (OR) with 95% confidence intervals (95%CI) were calculated according to Woolf's method. The software used was StatCalc program (Epi Info 2002; Centers of Disease Control and Prevention, Atlanta, GA, USA). For the haplotype analysis, pair-wise linkage disequilibrium measures were investigated and haplotypes were constructed using the expectation-maximization (EM) algorithm implemented in UNPHASED software [20]. *P *values below 0.05 were considered statistically significant. The power of the study to detect an effect of a polymorphism in disease susceptibility was estimated using the Quanto v 0.5 software (Department of Preventive Medicine University of Southern California, CA, USA).

## Results

Table 2 shows the allele and genotype distribution of the *RANTES*,*IL-8*, *IL-1α*, and *MCP-1 *polymorphisms. For all polymorphisms, the distribution of genotypes did not deviate from that expected from populations in Hardy-Weinberg equilibrium.

**Table 2:** Allele and genotype frequencies of *RANTES*, *IL-8*, *MCP-1 *and *IL-1α *polymorphisms in SLE patients and healthy controls.

	SLE patients		*Controls*		*P*	OR (95%CI)
*RANTES *-403	n	%	n	%		

Genotypes						
GG	369	73.8	333	69.3	0.1	
GA	113	22.6	135	28	0.04	0.75 (0.55–1.01)
AA	18	3.6	13	2.7	0.4	
Alleles						
G	851	85	801	83.3		
A	149	15	161	16.7	0.2	

*RANTES *R3	n	%	n	%		

Genotypes						
CC	326	73.8	340	77.6	0.06	
CT	104	23.5	90	20.6	0.3	
TT	12	2.7	8	1.8	0.4	
Alleles						
C	756	85.5	770	88		
T	128	14.5	106	12	0.1	

*IL-8 *-353	n	%	n	%		

Genoypes						
AA	126	28.7	125	30.3	0.6	
AT	215	49	194	47.1	0.5	
TT	98	22.3	93	22.6	0.9	
Alleles						
A	467	53.2	444	53.8		
T	411	46.8	380	46.2	0.7	

*IL-8 *+781	n	%	n	%		

Genotypes						
CC	164	35	143	33.3	0.6	
CT	238	51	221	51.5	0.8	
TT	65	14	65	15.2	0.6	
Alleles						
C	566	60.6	507	59.1		
T	368	39.4	351	40.9	0.5	

*IL-1α *-889	n	%	n	%		

Genotypes						
CC	220	52.7	209	49.7	0.4	
CT	164	39.3	166	39.5	0.9	
TT	33	7.9	45	10.7	0.2	
Alleles						
C	604	72.4	584	69.5		
T	230	27.6	256	30.5	0.2	

*MCP-1 *-2518	n	%	n	%		

Genotypes						
AA	238	57.2	250	58.5	0.6	
AG	173	35	154	36	0.7	
GG	39	7.8	23	5.4	0.1	
Alleles						
A	739	74.6	654	76.6		
G	251	25.4	200	23.4	0.3	

### *RANTES *typing

Genotyping of *RANTES *-403 G/A and R3 T/C was performed in 500 and 442 SLE patients and 481 and 438 healthy controls, respectively (table 2). No statistically significant differences were observed when the allele and genotype distribution was compared between SLE patients and healthy controls. Also, we found no association for the two marker haplotypes (table 3).

**Table 3:** Haplotype frequencies for *RANTES *and *IL-8 *polymorphisms in SLE patients and controls.

Gene	Haplotype	SLE patients	Healthy controls	*P*_*value*_	OR (95%CI)
RANTES					

	-403A/R3C	25 (5.7)	25 (5.8)	ns	
	-403A/R3T	50 (11.3)	40 (9.3)	ns	
	-403G/R3C	355 (80.7)	356 (83.4)	ns	
	-403G/R3T	10 (2.3)	6 (1.5)	ns	

*IL-8*					

	-353T/+781C	69 (8.6)	48 (6.2)	0.08	1.41 (0.94–2.10)
	-353T/+781T	316 (39.2)	303 (39.4)	ns	
	-353A/+781C	403 (50)	406 (52.7)	ns	
	-353A/+781T	18 (2.2)	13 (1.7)	ns	

### *IL-8 *typing

*IL-8 *-353 T/A and +781 C/T was genotyping in 439 and 467 SLE patients and 412 and 429 healthy controls, respectively for each polymorphism. We found a similar distribution in the allele and genotype frequencies between SLE patients and controls for both genetic variants. The haplotype estimation for the -353 T/A and +781 C/T *IL-8 *polymorphisms revealed a strong degree of linkage disequilibrium between the two variants (D' = 0.9) and showed a slight but non-significant increase of the -353T-+781C haplotype in SLE patients (8.5% vs 6.2%, *P *= 0.08, OR = 1.41, 95%CI = 0.94–2.10) (Table 3).

### *IL-1α *typing

*IL-1α *-889 was typing in 417 SLE patients and 420 healthy controls. We did not find any significant difference when allele and genotype frequencies were compared between SLE patients and healthy controls.

### *MCP-1 *typing

Table 2 show the allele and genotype distribution of the *MCP-1 *-2518 A/G polymorphism in 450 SLE patients and 427 controls. No significant differences in the allele and genotype frequencies of the *MCP-1 *-2518 A/G polymorphism were found between SLE patients and controls.

In addition, available clinical features of patients with SLE were analysed for possible association with the different alleles or genotypes of these polymorphisms. When we stratified SLE patients according to the presence of renal involvement, no statistically significant differences were observed in the distribution of *RANTES *-403, *RANTES *R3, *IL-1α *-889 and *MCP-1 *-2518 polymorphisms between SLE patients with and without lupus nephritis (table 4). Regarding *IL-8 *polymorphisms, the AT -353 genotype and the -353T/+781C haplotype showed an increased among lupus patients without nephritis compared with patients with nephritis (39.2% vs 49.4%, P = 0.03, OR = 0.66, 95%CI = 0.44–0.99 for AT -353 genotype) (5.7% vs 10%, P = 0.05, OR = 0.55, 95%CI = 0.28–1.05 for -353T/+781C haplotype) (table 4).

**Table 4:** Relationship between *RANTES*, *IL-8*, *MCP-1 *and *IL-1α *polymorphisms and the presence of nephritis in SLE Spanish patients.

	SLE with nephritis		SLE without nephritis		*P*	OR (95%CI)
*RANTES *-403	n	%	n	%		

Genotypes						
GG	136	73.5	230	73	0.9	
GA	44	23.8	71	22.5	0.7	
AA	5	2.7	14	4.4	0.3	
Alleles						
G	54	14.6	99	15.7		
A	316	85.4	531	84.3	0.6	

*RANTES *R3	n	%	n	%		

Genotypes						
CC	89	77.4	225	68.8	0.08	
CT	23	20	92	28.1	0.1	
TT	3	2.6	10	3	0.8	
Alleles						
C	201	87.4	542	82.9		
T	29	12.6	112	12.1	0.1	

*IL-8 *-353	n	%	n	%		

Genoypes						
AA	47	26.7	59	22.4	0.3	
AT	69	39.2	130	49.4	0.03	0.66 (0.44–0.99)
TT	60	34.1	74	28.2	0.2	
Alleles						
A	163	46.3	248	47.2		
T	189	53.7	278	52.8	0.8	

*IL-8 *+781	n	%	n	%		

Genotypes						
CC	74	39.6	99	35.3	0.3	
CT	85	45.4	151	54	0.07	
TT	28	15	30	10.7	0.2	
Alleles						
C	233	62.3	349	62.3		
T	141	37.7	211	37.7	0.9	

*IL8 *-353T/+781C						

Haplotypes						
-353T/+781C	15	5.7	39	10	0.05	0.55 (0.21–8.05)
-353T/+781T	104	39.7	149	38.2	0.7	
-353A/+781C	140	53.4	193	49.5	0.3	
-353A/+781T	3	1.2	9	2.3	0.3	

*IL-1α *-889						

Genotypes						
CC	72	49.3	138	50.9	0.7	
CT	59	40.4	115	42.4	0.7	
TT	15	10.3	18	6.7	0.2	
Alleles						
C	203	69.5	391	72.1		
T	89	30.5	151	27.9	0.4	

*MCP-1 *-2518						

Genotypes						
AA	86	54.4	170	58.2	0.4	
AG	61	38.6	100	34.2	0.3	
GG	11	7	22	7.5	0.8	
Alleles						
A	233	73.7	440	75.3		
G	83	26.3	144	24.7	0.6	

Similarly, no significant differences were observed between all these genetic variants and the following variables: sex, age at onset, articular involvement, cutaneous lesions, photosensitivity, hematological alterations, neurological disorders and serositis (data not shown).

## Discussion

In this work, we have tested six functional polymorphisms of four strong candidate genes for association with SLE. No evidence of association was detected for *RANTES *(-403 G/A, R3 T/C),*IL-8 *(-353 A/T, +781 C/T), *IL-1α *(-889C/T), and *MCP-1 *(-2518 G/A) polymorphisms. However, a significant association was observed for the *IL-8 *haplotype with SLE nephritis, which cannot be considered as significant after correction for multiple comparisons.

All these genes have been previously associated with susceptibility and development to several autoimmune disorders, included SLE [16,21-27]. For example, recent studies in Asian populations found another *RANTES *polymorphism (-28C/G) to be associated with increased risk of developing SLE, but failed to detect any association of *RANTES *-403 polymorphisms with SLE [22,23]. We did not test the -28C/G variant as -28G allele is relatively uncommon in Caucasians [28].

The genetic variant *IL-8 *-845C showed a high association to severe lupus nephritis (LN) in an African American population [16], but also this allele has a very low frequency in Caucasian populations [16,29]. The trend of association that we have found between the haplotypes and LN and the reported association of other *IL-8 *variants this African American population, shows that variants in this chemokine may have a minor influence on the risk of developing nephritis in SLE patients.

Similar observation could be made for the reported association of the *IL-1α *-889C/T variant to SLE in a White and African American populations from United States, which we failed to replicate [30]. With regard to the *MCP-1 *-2518 polymorphism, an American study showed that an A/G or G/G genotype may predispose to the development of SLE and further indicated that SLE patients with these genotypes may be at higher risk of developing LN [3].

The fact that we do not observe an association and fail to confirm some previous studies may be caused by a Type II error (false-negative). This is however unlikely because our sample has more than 80% power to detect the relative risk similar to the other studies at the 5% significance level. Furthermore, the genotype frequencies did not differ from Hardy-Weinberg expectations, and allele and genotype frequencies in our Spanish population are similar to those reported previously in other Caucasian populations [16,26,31,32]. The failure to replicate reported associations is a common event in the search for genetic determinants of complex diseases, due either to genuine population heterogeneity or a different sort of bias [33]. The lack of replication in our population may alternatively be explained by a different racial composition of that study from ours, or that presence of environmental factors to which the Asian, American, and African populations, but not the Spanish population, are exposed. In addition, genetic differences are known to exist between the different ethnic groups, such as, African American and Caucasians.

## Conclusion

In conclusion, our results suggest that functional genetics variation in *RANTES*, *IL-8*, *IL-1α*, and *MCP-1 *do not play a major role in SLE susceptibility in the Spanish population.

## Competing interests

The author(s) declare that they have no competing interests.

## Authors' contributions

ES carried out the genotyping and statistical analysis and drafted the manuscript, JMS collected the samples, JLC collected the samples, EDR collected the samples, RGP collected the samples, FJGH collected the samples, JJA collected the samples, MFGE collected the samples, JM participated in the manuscript design and coordination and helped to draft the manuscript, BK participated in the manuscript design, reviewed the statistical analysis and helped to draft the manuscript.

## Pre-publication history

The pre-publication history for this paper can be accessed here:


